# MiaA (Rv2727c) mediated tRNA isopentenylation of *Mycobacterium tuberculosis *H37Rv

**DOI:** 10.22099/mbrc.2022.43197.1726

**Published:** 2022-06

**Authors:** Smitha Soman, Siya Ram

**Affiliations:** 1 School of Biotechnology, Gautam Buddha University, Gautam Budh Nagar, Greater Noida, Uttar Pradesh, India; 2 School of Sciences, Indira Gandhi National Open University, Maidan Garhi, New Delhi, India

**Keywords:** Tuberculosis, M. smegmatis, MiaA, i6A, DMAPP, Isopentenyl transferase

## Abstract

tRNA modifications play a significant role in the structural stability as well as translational fidelity in all organisms from bacteria to humans. They also play a major role in bacterial physiology by regulating translation in response to various environmental stresses. Modifications coming at the anticodon-stem loop (ASL) are particularly important as they stabilize codon-anticodon interactions, ensuring accuracy and speed in decoding mRNAs Addition of isopentenyl group (i6A) at A37 position by tRNA isopentenyltransferase (MiaA) is a well conserved modification from bacteria to human. We studied *M. tuberculosis* MiaA from strain H37Rv and identified the target tRNAs for this modification based on the A36A37A38 motif. i6A modification of target tRNAs tRNA^Leu^CAA, tRNA^Phe^GAA, tRNA^Trp^CCA and tRNA^Ser^CGA were further confirmed by isopentenyltransferase assay providing the substrate DMAPP and recombinant MiaA enzyme.

## INTRODUCTION

Tuberculosis, caused by *Mycobacterium tuberculosis* (M. tb), is the leading cause of death worldwide from a single infectious agent, only second to COVID 19 [1]. Even though TB is a preventable and curable disease, factors such as lack of an effective vaccine, emergence of multidrug as well as extensive drug resistant strains, HIV-TB co-infection, and a complex drug regime hamper the control of this ancient disease [2]. M. tb is a slow growing bacterium that transmits through aerosol from an infected person to a healthy individual. Depending on the innate immune response of the host and virulence of the organism, M. tb either starts multiplying leading to active infection or gets contained in granuloma. Granuloma formation leads to the latency stage of the infection where the bacteria stays in a dormant, asymptomatic form, but cannot be eliminated from the host [3].

A deeper understanding of the biology of the organism is needed to develop new antimicrobial drugs as well as vaccines to control the spread of this disease. Being an intracellular pathogen, M. tb must overcome a variety of host induced stress conditions to be a successful pathogen. Identifying and targeting the specific pathways that enable the pathogen to avoid various physiological stress including low pH, high oxidative stress, and starvation will enable us to develop novel therapeutics. 

tRNA modifications are one way to regulate translation of proteins under various stress conditions in bacteria. tRNAs, which act as adaptor molecules during protein synthesis by decoding the codon sequence of mRNA and providing the right amino acid to the peptide chain, are heavily modified post-transcriptionally. While some modifications increases the structural integrity of the tRNA, modifications in the anticodon stem loop (ASL) increases translational fidelity. It is observed that certain tRNA modifications shift dynamically, equipping the bacteria to adapt well with changing physiological conditions like oxidative stress, starvation, hypoxia etc. [4]. Hypoxia induced tRNA modifications are already reported to play a role in stress response in *Mycobacterium bovis* BCG [5]. 

Isopentenyladenosine (i6A) at position 37 is one of the first modified bases identified in tRNA and is present in all organisms from bacteria to humans except archaea [6]. This modification stabilizes codon-anticodon interaction, thereby increasing translational speed and fidelity [7]. In *E.coli*, MiaA catalyses the transfer of an isopentenyl group from dimethylallylpyrophosphate (DMAPP) to N6 position of adenosine, which is essential for the transition of the bacteria to stationary phase [8]. MiaA is also associated with virulence in pathogenic bacteria like *Shigella flexneri*, where mutation in the gene affected expression of many virulence genes [9]. In this study we are looking into the tRNA isopentenyltranferase (MiaA) of *Mycobacterium tuberculosis* strain H37Rv. We identified the tRNA targets of i6A modification in M. tb and isopentenlyation of tRNAs were confirmed by providing the substrate DMAPP and recombinant MiaA.

## MATERIALS AND METHODS

**Bacterial strain and culture conditions:** *Mycobacterium smegmatis*, mc2155, was cultured on Middlebrook 7H10 agar supplemented with 10% OADC or 7H9 broth with 10% ADC and 0.1% Tween-80. After transformation, Kanamycin was added at 20 µg/ml to the growth media for positive selection. Isovaleronitrile (IVN) was purchased from Sigma and 1M stock was prepared in DMF.

**Generation of expression vector pNit3xFLAG-miaA:** M. tb H37Rv DNA, *E.coli*- Mycobacteria shuttle plasmid pNit3xFLAG vector and antibodies were obtained from Dr. Vinay Nandicoori’s Lab, National Institute of Immunology, New Delhi, India. 945bp miaA gene (Rv2727c) was PCR amplified from H37Rv with restriction sites *Nde*I and *Hind*III and was ligated to the *Nde*I-*Hind*III site of pNit3xF. pNit3xF-miaA plasmid was electroporated into electrocompetent *M. smegmatis* at 25kV, 25mF, with the pulse controller resistance of 1000 in an electroporation chamber and the cells were plated on 7H10 agar with kanamycin[10].

**Expression M. tb MiaA from** ***Mycobacterium smegmatis*** **and Western blot:** *M. smegmatis* transformed with pNit3XF-miaA was inoculated at 0.025 OD in 7H9 broth with kanamycin and protein production was induced by adding 5μM IVN. *M. smegmatis *pNit3XF without insert was also included as control. The cultures were grown at 37^o^C for 16hr, and were centrifuged at 4000 rpm for 5 min. The pellets were resuspended in PBSG (PBS containing 5% glycerol) at 1:3 (pellet weight in g:PBSG volume in ml) ratio. Cells were lysed using 0.1mm zirconium beads (Biospec) for ten 1 min cycles with 2 min intervals on ice using Biospec mini bead-beater. The lysates were clarified at 13,000 rpm at 4^o^C and the protein concentration was estimated using Pierce^TM ^BCA protein assay kit. MiaA expression was confirmed by Western blot. For this, 40μg whole cell lysate was separated on 12% SDS-PAGE and transferred to nitrocellulose membrane, blocked with 5% BSA, followed by overnight probing with anti-FLAG M2 (Sigma-F1804) antibody at 1:2500 dilution and anti- GroEL1 antibody (raised in rabbit, 1:10,000 dilution) [11].

***In vitro*** **transcription of i6A target tRNAs of M. tb:** The target tRNA genes were PCR amplified from H37Rv DNA with a T7 promoter sequence at the 5’ end (5’TAATACGACTC ACTATAGG3’). *In vitro* transcription of these tRNAs were done at 37^o^C, providing T7 RNA Polymerase, NTP mix (5mM of ATP, GTP, CTP & UTP), α^32^P GTP along with the PCR products for 3hr.

**Immunoprecipitation of M. tb MiaA and tRNA isopentenyltransferase assay:** 1mg of whole cell lysate (WCL) of *M. smegmatis* was used for the immunoprecipitation (IP) of MiaA. FLAG-M2 affinity gel beads washed with TBS (50mM Tris HCl, 150mM NaCl, pH 7.4) were incubated with WCL overnight at 4^o^C and MiaA was eluted with 0.1M glycine (pH3.5), followed by neutralization of the elute with 1/10th volume 1M Tris-HCl (pH 7.4). Western blotting was done to confirm MiaA (38kD), followed by protein estimation by BCA kit. Isopentenyl transferase assay of the target tRNAs were done by incubating ^32^P labelled tRNA with MiaA in the presence or absence of the substrate dimethylallylpyrophospate (DMAPP- Sigma) at 37^o^C for 1hr [12].

## RESULTS

**Figure 1:**
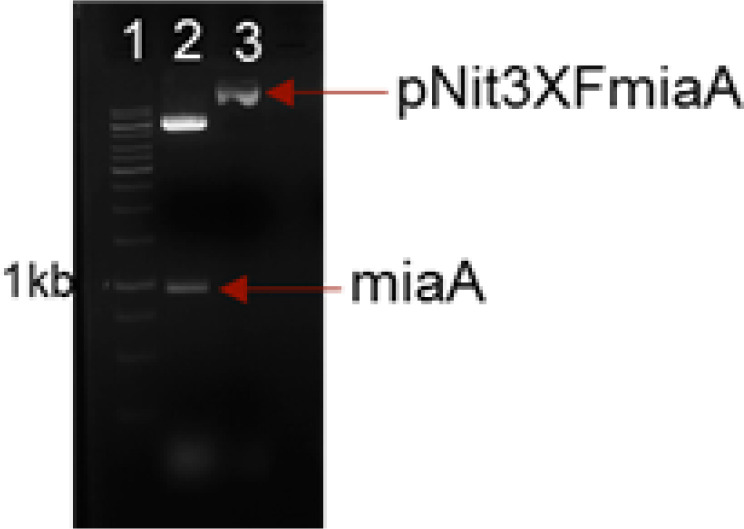
*Nde*I-*Hind*III digestion of pNit3XFLAG miaA. Lane1-1kb DNA ladder (Thermo), 2-*Nde*I-*Hind*III digested pNit3xFmiaA, 3-undigested pNit3xFmiaA

**Figure 2:**
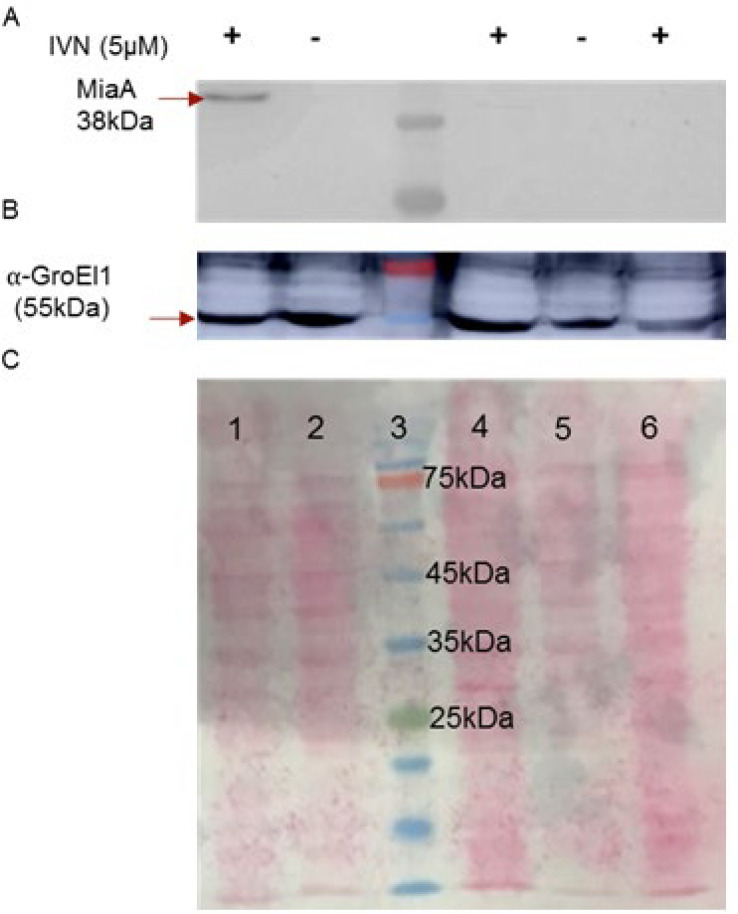
Expression of M. tb MiaA from *M. smegmatis *WCL of  *M. smegmatis *probed with **A-**Western Blot probed with ⍺-FLAG Ab, **B**-⍺-GroEl1 Ab, **C-**Ponceau stained blot Lane 1-pNit3XFmiaA +IVN, 2-pNit3XFmiaA without IVN, 3-Prestained Protein Marker (Thermo), 4- pNit3XF +IVN, 5- pNit3XF without IVN, 6- *M. smegmatis *Control

Cloning and expression of M. tb MiaA. To purify M. tb MiaA, we amplified the miaA gene (Rv2727c) using gene specific primers from M. tb H37Rv genomic DNA and cloned into pNit3xFLAG vector. The clones were screened for insert release (Fig. 1) and confirmed by sequencing. The insert was of 945bp. To check expression of the FLAG tagged MiaA, *M. smegmatis* was transformed with pNit3XF-miaA plasmid followed by induction with 5μM IVN. After induction, lysates were prepared followed by western transfer (Fig. 2C) and probing with α-FLAG M2 antibody. We could identify a band corresponding to the size of MiaA (38kDa) (Fig. 2A). The induction condition of 5μM IVN for 16hr at 37^o^C was found to be optimal. *M. smegmatis* and *M. smegmatis* with pNit3XF, used as controls, didn’t show any bands as expected. α-GroEL1 was used as the loading control (Fig. 2B).

**Figure 3:**
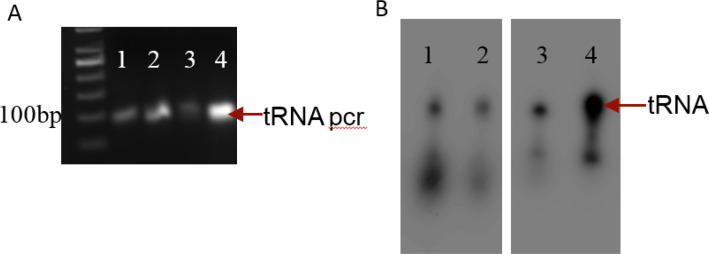
*In vitro *transcription of MiaA target tRNAs. **A)**: PCR amplification of tRNA genes from H37Rv. Lane 1-LeuCAA, 2-SerCGA, 3-PheGAA, 4-TrpCCA **B)**: Autoradiograph showing *in vitro *synthesized target tRNAs Lane 1-tRNA^Leu^CAA, 2-tRNA^Ser^CGA, 3-tRNA^Phe^GAA, 4-tRNA^Trp^CCA

Identification of MiaA tRNA targets in M. tb. i6A modification by MiaA requires the motif A36A37A38 in the tRNA stem-loop [13]. In bacteria, Leu, Phe, Ser, Cys, Trp and Tyr tRNAs have codons with U in the first position. We analysed the tRNA sequences of all 45 tRNAs in M. tb and identified eight tRNAs with A36A37A38 motif (Table 1). In order to confirm MiaA activity, we amplified these tRNA genes from M. tb genome (Fig. 3A). T7 promoter sequence included at the 5’ end of each forward primer enabled the *in vitro* transcription of the target tRNAs with T7 RNA polymerase. The tRNAs were internally labelled with 32P to visualize them by autoradiograph (Fig. 3B).

**Table1:** Target tRNAs of i6A modification in M.tb, codons decoded and genes encoding these tRNAs

**Target tRNAs of i6A modification in ** ***M. tuberculosis***	**Codons decoded**	**tRNA gene in H37Rv**	**Gene length**
Cys_GCA	UGC, UGU	cysU	71bp
Leu_CAA	UUG	leuV	74bp
Leu_TAA	UUA	leuX	74bp
Phe_GAA	UUC, UUU	pheU	74bp
Ser_CGA	UCG	serX	87bp
Ser_TGA	UCA	serU	87bp
Trp_CCA	UGG	trpT	73bp
Tyr_GTA	UAC, UAU	tyrT	81bp

**Figure 4:**
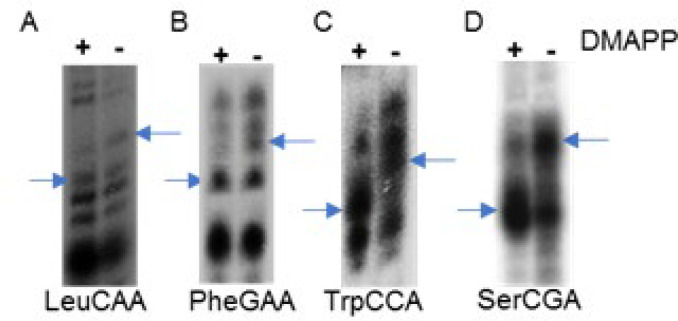
Isopentenyl transferase assay. i6A target tRNAs of M. tb tRNA^Leu^CAA, tRNA^Phe^GAA, tRNA^Trp^CCA and tRNA^Ser^CGA were labeled internally with ^32^P-Guanosine and incubated with immunoprecipitated MiaA in the presence or absence of the substrate DMAPP at 37^o^C. 4A-D: tRNA fragment showing shift in migration indicating addition of i6A group. Shifted bands with A36A37A38 motif after T1 digestion are shown by the arrow, LeuCAA ACUCAAAACCCG, 12nt; PheGAA: AAAAG ,5nt; TrpCCA: UCUCCAAAACCG, 12nt; SerCGA: AAAG, 4nt

tRNA isopentenyltransferase activity of M. tb MiaA: In all organisms with i6A modification the isopentenyl group comes from the substrate dimethylallylpyrophospate (DMAPP). *E.coli* and *M. tuberculosis* synthesize DMAPP through 2C-methyl-D-erythritol 4-phosphate (MEP) pathway whereas eukaryotes and some other bacteria utilize mevalonate pathway [14]. Target tRNAs tRNA^Leu^CAA, tRNA^Phe^GAA, tRNA^Trp^CCA, tRNA^Ser^CGA were incubated with 5.3M MiaA and 0.2mM DMAPP and each tRNA was precipitated after one hour of modification reaction. tRNA without DMAPP served as control. tRNA pellets resuspended in 8M Urea were digested with RNase T1, which cleaves at the 3’ end of all guanosine in the tRNA. The digested tRNA fragments were separated on 20% polyacrylamide denaturing gel. Addition of i6A group to the tRNA fragment at A37 causes a mobility shift of that fragment, by comparing tRNA fragments in the presence and absence of DMAPP, this can be identified. The RNA fragment length with i6A modification varied with each tRNA depending on the position of guanosines in the RNA sequence. The target fragment with i6A modification in tRNA^Leu^CAA and tRNA^Trp ^CCA were 12 nucleotide, tRNA^Phe^ GAA was 5 nucletide and tRNA^Ser^ CGA, 4 nuclotide. Since M. tb genome is G-C rich, T1 digestion produces smaller nucleotide products. As a result, signal intensity was higher with lower nucleotide bands, which were also somewhat diffused. This made it difficult to differentiate shift in bands when the target fragments are small. Still, a shift in the position of these fragments could be clearly observed (Fig. 4A-D) in comparison with tRNA fragments without substrate, indicating that the MiaA enzyme of M. tb could modify these tRNAs. 

## DISCUSSION

i6A modification is present universally from prokaryotes to eukaryotes and the tRNA isopentenyltransferase genes MiaA (bacteria), Mod5, Tit1 (yeast), GRO-1 (nematodes) and TRIT1 (mammals) are homologous and are conserved through evolution [6]. Bacterial and mitochondrial i6A37 can be further modified into 2-methylthio-N6-isopentenyl-A37 (ms2i6A37) by MiaB and Cdk5 regulatory subunit associated protein1 (Cdk5rap1) respectively [15]. Methylthiolation can only happen to those tRNAs which are already i6A modified; without this modification, mitochondrial tRNAs are sensitive to oxidative stress which leads to mitochondrial diseases [16]. Mice lacking this modification is reported to have myopathy and cardiac dysfunction under stress conditions due to defective mitochondrial translation. A homozygous p.Arg323Gln mutation in TRIT1 in humans cause decreased mitochondrial expression and activity leading to neurodevelopmental disease [17,18]. 

In bacteria, i6A modification plays a role in fitness and virulence and can affect the metabolic and stress response when the organism moves from one physiological state to another. But the role of this modification can change from one species to another and can be deduced only at a given context. For example, in *E.coli* stress transcription factor RpoS needs i6A modified tRNAs for its translation as the RpoS mRNA is enriched with LeuUUA codons. So lack of i6A modification impairs the successful transition of *E.coli* to stationary phase [8,19]. But in Extraintestinal Pathogenic *E.coli* (ExPEC), miaA is found essential for the gut colonization; miaA mutant strains have limited metabolic flexibility and virulence in comparison with the wild type. They also exhibit limited tolerance to oxidative, nitrosative and osmotic stresses[20]. In the pathogen *Shigella flexneri*, MiaA is essential for the expression of many virulence genes required for the invasion and spreading of the bacteria leading to shigellosis. Introduction of miaA mutation resulted in avirulent phenotypes with tenfold reduction in the expression of virulence associated virF gene and hemolytic activity in many pathogenic Shigella sp. In all these cases the mRNA levels of the virulence genes remain unchanged, suggesting the regulation happening in the translational level [9].

Considered as one of the most successful pathogens, M. tb avoids host induced stress conditions like nutrient deprivation, nitrogen and oxygen free radicals, acidic pH, hypoxia etc. Studies in *M. bovis* BCG showed that there is a distinct change in the tRNA modification pattern when the bacteria enters and exits hypoxia. This resulted in biased translation of those genes that are enriched with codon requiring modified tRNAs. Many of these genes were part of Dos regulon which controls early hypoxic response in Mycobacteria [5]. Regulatory role of other tRNA modifications in M. tb survival and virulence is not yet known. Role played by MiaA in the stress response as well as pathogenesis in other bacteria suggest it may play more than a housekeeping role in M. tb also. The results provided here clearly shows that tRNA^Leu^CAA, tRNA^Phe^GAA, tRNA^Trp^CCA, tRNA^Ser^CGA are eligible targets for i6A modification. Since i6A is sourced from DMAPP, which is an intermediate of important pathways, this modification could also be a link that connects metabolism with tRNA modifications and protein synthesis. Further studies with gene knockouts of MiaA and identifying the target mRNAs that are affected by this tRNA modifications will provide more insights into the biology of M. tb and thereby help in disease control. Since translation is a process that is targeted by mechanisms that rapidly regulate gene expression in response to stress conditions, its detailed understanding will enable us to address the proliferation of bacteria in host cells.

## Conflict of Interest

The authors declare no conflict of interest.
